# Role of extracorporeal membrane oxygenation in pediatric cancer patients: a systematic review and meta-analysis of observational studies

**DOI:** 10.1186/s13613-022-00983-0

**Published:** 2022-01-29

**Authors:** Valerie Slooff, Rianne Hoogendoorn, Jeppe Sylvest Angaard Nielsen, John Pappachan, Angela Amigoni, Fabio Caramelli, Omer Aziz, Enno Wildschut, Sascha Verbruggen, Roman Crazzolara, Christian Dohna-Schwake, Jenny Potratz, Jef Willems, Judit Llevadias, Andrea Moscatelli, Alessia Montaguti, Gabriella Bottari, Matteo Di Nardo, Luregn Schlapbach, Roelie Wösten-van Asperen, the POKER (PICU Oncology Kids in Europe Research group) research consortium of ESPNIC (European Society of Paediatric Neonatal Intensive Care)

**Affiliations:** 1 Department of Pediatric Intensive Care, University Medical Centre Utrecht/Wilhelmina Children’s Hospital, Lundlaan 6, 3584 EA Utrecht, The Netherlands; 2 Department of Neonatology and Pediatric Intensive Care, Rigshospitalet, Copenhagen, Denmark; 3 Pediatric Intensive Care Unit, University Hospital Southampton, Southampton, UK; 4 Pediatric Intensive Care Unit, Department of Woman’s and Child’s Health, Padua University Hospital, Padua, Italy; 5 Department of Woman, Child and Urological Diseases, Pediatric Intensive Care Unit, University-Hospital S. Orsola-Malpighi Policlinic, Bologna, Italy; 6 Department of Pediatric Intensive Care, Royal Bristol Children’s Hospital, Bristol, UK; 7 Intensive Care, Department of Pediatrics and Pediatric Surgery, Erasmus Medical Centre, Sophia Children’s Hospital, Rotterdam, The Netherlands; 8 Department of Pediatrics, Pediatric Intensive Care Unit, Medical University of Innsbruck, Innsbruck, Austria; 9 Department of Pediatric Intensive Care, Universitätsklinik Essen, Essen, Germany; 10 Department of General Pediatrics-Intensive Care Medicine, University Children’s Hospital Münster, Munster, Germany; 11 Department of Pediatric Intensive Care, Ghent University Hospital, Ghent, Belgium; 12 Department of Pediatric Intensive Care, Newcastle Upon Tyne Hospitals NHS Foundation Trust, Newcastle upon Tyne, UK; 13 Department of Pediatric Intensive Care, Gaslini Hospital, Genova, Italy; 14 Pediatric Intensive Care Unit, Ospedale Pediatrico Bambino Gesù, IRCC, Rome, Italy; 15 Pediatric and Neonatal Intensive Care Unit, Children’s Research Centre, University Children’s Hospital Zurich and University of Zurich, Zurich, Switzerland

**Keywords:** Extracorporeal membrane oxygenation, Pediatric intensive care unit, Cancer, Outcomes, Systematic review, Meta-analysis

## Abstract

**Background:**

The use of extracorporeal membrane oxygenation (ECMO) in pediatric patients with underlying malignancies remains controversial. However, in an era in which the survival rates for children with malignancies have increased significantly and several recent reports have demonstrated effective ECMO use in children with cancer, we aimed to estimate the outcome and complications of ECMO treatment in these children.

**Methods:**

We searched MEDLINE, Embase and CINAHL databases for studies on the use ECMO in pediatric patients with an underlying malignancy from inception to September 2020. This review was conducted in adherence to Preferred Reporting Items for Systematic Review and Meta-Analysis statement. Study eligibility was independently assessed by two authors and disagreements resolved by a third author. Included studies were evaluated for quality using the Newcastle–Ottawa Scale (NOS). Random effects meta-analyses (DerSimonian and Laird) were performed. The primary outcomes were mortality during ECMO or hospital mortality.

**Results:**

Thirteen retrospective, observational cohort studies were included, most of moderate quality (625 patients). The commonest indication for ECMO was severe respiratory failure (92%). Pooled mortality during ECMO was 55% (95% confidence interval [CI], 47–63%) and pooled hospital mortality was 60% (95% CI 54–67%). Although heterogeneity among the included studies was low, confidence intervals were large. In addition, the majority of the data were derived from registries with overlapping patients which were excluded for the meta-analyses to prevent resampling of the same participants across the included studies. Finally, there was a lack of consistent complications reporting among the studies.

**Conclusion:**

Significantly higher mortalities than in general PICU patients was reported with the use of ECMO in children with malignancies. Although these results need to be interpreted with caution due to the lack of granular data, they suggest that ECMO appears to represents a viable rescue option for selected patients with underlying malignancies. There is an urgent need for additional data to define patients for whom ECMO may provide benefit or harm.

**Supplementary Information:**

The online version contains supplementary material available at 10.1186/s13613-022-00983-0.

## Introduction

The prognosis of children with malignancies has improved significantly over the past two decades. Precision medicine and immunotherapy along with other new therapies have translated into improved survival and cure rates. Currently, 5-year all-cancer survival is almost 80% in children [1]. However, intensified treatment protocols have resulted in more complications, leading to a growing number of children requiring admission to a pediatric intensive care unit (PICU). In this context, identifying patients who are most likely to benefit from invasive organ support is crucial. Defining the optimal timing of these life-sustaining therapies is one of the top five research priorities identified by a recent Delphi survey among pediatric intensivists and oncologists [2].

Patients who fail conventional pulmonary and/or circulatory support may be considered for extracorporeal membrane oxygenation (ECMO). The use of ECMO in children with underlying malignancies has historically been contraindicated; however, several recent reports have demonstrated its effectiveness in selected cases. In addition, children with malignancies are becoming more prevalent in the Extracorporeal Life Support Organization (ELSO) registry which collates data from 422 pediatric ECMO centers worldwide [3]. The indications for ECMO are expanding, but is effectiveness and the rate of associated complications in high-risk populations such as children with malignancy is poorly described. Most of the existing data come from small, single-center retrospective studies.

Given the controversial nature of this use of ECMO, this systematic review and meta-analysis was designed to describe the characteristics of children with an underlying malignancy receiving ECMO and the complications and survival rates observed.

## Methods

### Search strategy and selection criteria

We conducted a systematic review and meta-analysis using the Preferred Reporting Items for Systematic Reviews and Meta-analyses (PRISMA) recommendations (Additional file 1: Table S1) [4]. It was anticipated that the majority of studies would be observational, so we followed the guidelines of reporting systematic reviews and meta-analysis of observational studies [5, 6].

Two investigators (VS and RH) performed a comprehensive literature search of MEDLINE, Embase, and CINAHL databases from inception to September 30, 2020. Databases were electronically searched for relevant publications using combinations of the following medical subject headings (MeSH) and keywords “Extracorporeal membrane oxygenation” AND “Neoplasms” AND “Pediatric” (Additional file 1: Table S2). In addition, a hand search of the references in included full texts was performed to identify additional articles for inclusion.

Articles were included if they reported outcomes for pediatric oncology patients who were supported on ECMO. If a study also included non-oncology patients, but a clearly defined subset of pediatric oncology patients was described, we included the study, but only collected data from these oncology patients. In addition, for studies that included overlapping patients (period of overlap > 1 year) from the same registry, the largest study was included in the meta-analysis, while the rest of the studies were included in the review. Only studies published in English were considered for inclusion. We excluded studies that included only adult patients, review articles, conference proceedings, correspondence, case reports, publications in abstract form only, and editorials. In addition, for studies including children supported by ECMO following hematopoietic stem cell transplantation (HSCT), only those in which data on HSCT performed for malignant diseases was reported were included.

### Data extraction and quality analysis

Abstracts of studies identified were screened, and those that met the inclusion criteria underwent full-text review by two independent investigators (VS and RH). Disagreements were reviewed by a third reviewer (RW), who had a deciding vote. Data for study design, patient characteristics, interventions, and study outcomes were abstracted independently and in duplicate.

The quality of the included studies was assessed by using the Newcastle–Ottawa Scale for observational studies [7, 8]. The scale evaluates three domains of bias: selection of participants (i.e., representativeness of the cohorts, ascertainment of exposure, and outcome of interest not being present at the start of study), comparability (to account for confounders that might influence the outcome of interest), and measure outcomes (i.e., methods for outcome assessment, appropriateness of the length of time to assess the outcome, and losses to follow-up that might compromise validity). This scale consists of a grading system with a maximum score of 9. A score of ≥ 7 points indicates that a study is of high quality [7, 8]. We used the Grading of Recommendations, Assessments, Developments and Evaluations (GRADE) system to assess the certainty of evidence [9, 10].

### Outcomes

The primary outcome was mortality on ECMO and at hospital discharge. Secondary outcomes included duration of ECMO and complications while on or associated with ECMO.

### Data analysis

Descriptive statistics were reported as medians and IQRs for continuous variables and counts and percentages for categorical variables. As we anticipated considerable between-study heterogeneity, an inverse-variance weighted random-effects model was used to pool effect sizes as suggested by DerSimonian and Laird [11]. Mortality outcomes were presented as proportions. In the case of overlapping patient data, we included the largest study and excluded overlapping studies in the meta-analysis.

Heterogeneity among studies was assessed with the *I*^2^ measure, where *I*^2^ > 50% suggests substantial heterogeneity [12, 13]. We subsequently performed sensitivity analyses to determine the influence of individual studies on the overall effect, including a leave-one-out analysis which iteratively removed one study at a time, generating Baujat plots, and influence diagnostics [14, 15]. Publication bias was assessed by constructing a funnel plot and by using the Egger’s test [16].

All statistical analyses were performed in R studio (Version 4.0.5, R studio, Inc. Boston) using the ‘meta’ and ‘dmetar’ packages [17, 18].

## Results

**Fig. 1:**
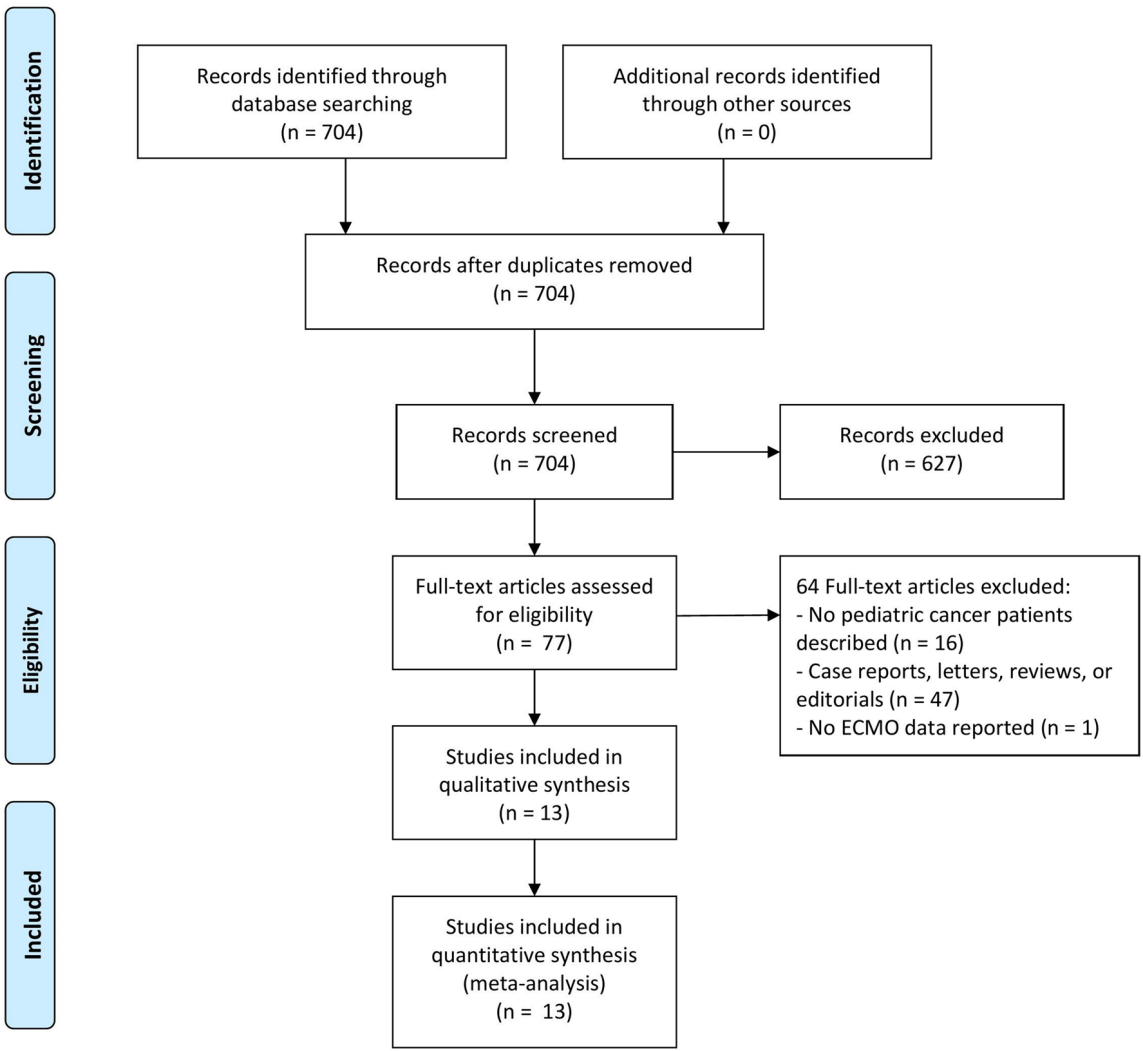
Flowchart of study selection

Our search retrieved 704 citations, 77 of which were selected for full-text review (Fig. 1). Thirteen studies with a combined population of 625 patients fulfilled our inclusion criteria—all were observational, retrospective studies (Table 1) [19–31]. Four studies, all from the ELSO registry, had potential overlapping information, although inclusion criteria varied between the studies [20, 21, 23, 25]. Eleven studies reported the underlying oncological diagnosis, with a predominance of hematological malignancies (Additional file 1: Table S3) [19–24, 26–30]. Four studies included HSCT patients [20, 24, 27, 28]; however, none of these studies specified the characteristics of this subgroup separately. One study included exclusively HSCT patients [23]. Studies varied with respect to criteria for initiation of ECMO, but the commonest indication was respiratory failure. The number of patients ranged from 4 to 200 per study. Six studies included patients with non-respiratory indications for the initiation of ECMO, including sepsis (*n* = 9), cardiac failure (*n* = 17), and extracorporeal cardiopulmonary resuscitation (*n* = 10) (Additional file 1: Table S4) [21, 23, 24, 27–29].

**Table 1:** Characteristics of included studies

Study	Country	Period	Study design	Inclusion criteria	ECMO group of children with underlying malignancies
Lindén et al. 1999 [19]	Sweden	NR	Retro, single center	Underlying malignancy and PCP pneumonia	4
Gupta et al. 2008 [20]	ELSO registry	1985–2004	Retro, multicenter ELSO registry	Immunocompromised condition and respiratory failure as indication for ECMO Age 30 days–19 yrs	60
Gow et al. 2009 [21]	ELSO registry	1992–2007	Retro, multicenter ELSO registry	Underlying malignancy Age < 21 yrs	107
Meister et al. 2009 [22]	Austria	NR	Retro, single center	Leukemia	4
Di Nardo et al. 2014 [23]	ELSO registry	1991–2012	Retro, multicenter ELSO registry	HSCT patients Age < 18 yrs	15
Smith et al. 2016 [24]	Australia	1993–2014	Retro, single center	Pediatric cancer patients with neutropenic sepsis	9
Bailly et al. 2017 [25]	ELSO registry	2001–2013	Retro, multicenter ELSO registry	ECMO for respiratory failure due to a primary pulmonary diagnosis Age 7 days–18 yrs	161
Cortina et al. 2018 [26]	Austria	2004–2007	Retro, single center	Leukemia	9
Maue et al. 2019 [27]	USA	2011–2016	Retro, single center	Pediatric oncology and/or HSCT patients	5
Steppan et al. 2020 [28]	USA	2011–2018	Retro, multicenter PEDECOR registry	Pediatric oncology and/or HSCT patients	16
Ranta et al. 2020 [29]	Sweden	2008–2016	Retro, multicenter	Hematological malignancies	12
Coleman et al. 2020 [30]	USA	2004–2013	Retro, multicenter PHIS registry	Underlying malignancy, genetic disorders or high-risk congenital heart disease	200
Friedman et al. 2020 [31]	USA	2011–2016	Retro, multicenter	Patients requiring VV-ECMO Age 14 days-18 yrs	23

*ECMO* extracorporeal membrane oxygenation, *ELSO registry* extracorporeal life support organization registry including > 145 centers worldwide, *HSCT* hematopoietic stem cell transplantation, *PCP* *Pneumocystis carinii* pneumonia, *Retro* retrospective, *yrs* year

### Primary outcome: ECMO and hospital mortality

**Fig. 2:**
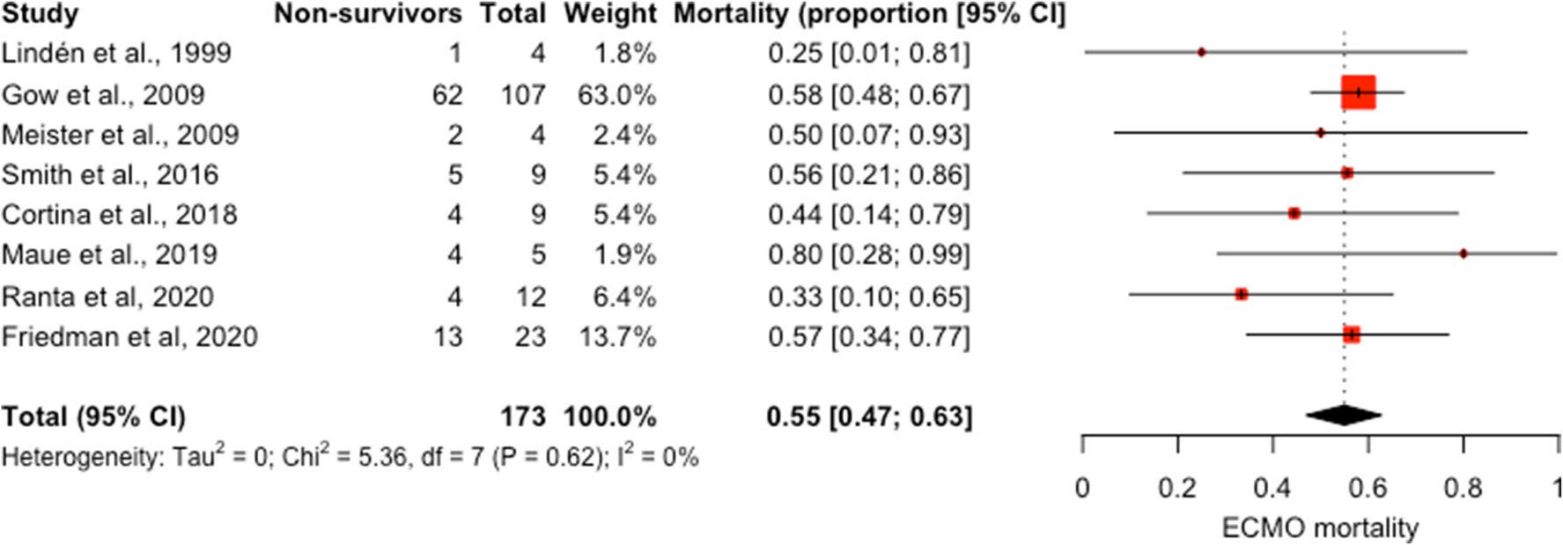
Forest plot of mortality on ECMO for pediatric cancer patients using a random-effects model. Individual mortality for each study and the pooled weighted estimate shown with 95% confidence intervals (CI). Vertical dotted line represents the pooled weighted estimate

Nine studies reported mortality during ECMO [19, 21–24, 26, 27, 29, 31]. Two studies retrieved their data from the ELSO registry with overlapping study periods [21, 23]. We included the largest study for the meta-analysis [21]. When data from the studies were pooled, mortality during ECMO among 173 patients was 55% (95% CI 47–63%) (Fig. 2). There was low heterogeneity among the studies (*I*^2^ 0%); however, the confidence interval was large (95% CI 0.0–67.6). Influence analysis revealed one study that was influential [21] (Additional file 1: Figure S1). After excluding this study, the remaining eight studies had 59 patients with a cumulative pooled mortality of 49% (95% CI 35–63%) (Additional file 1: Figure S2 and Table S5).

**Fig. 3:**
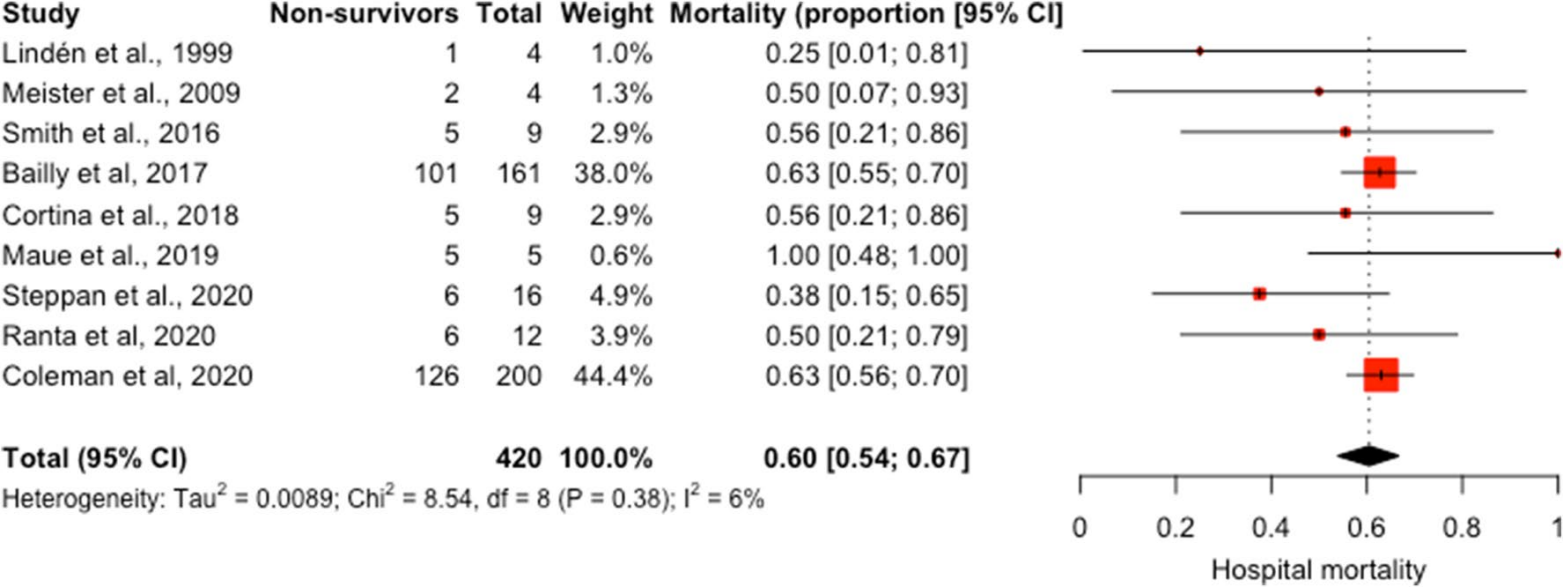
Forest plot of hospital mortality for pediatric cancer patients who were supported on ECMO. Individual mortality for each study and the pooled weighted estimate shown with 95% confidence intervals (CI). Vertical dotted line represents the pooled weighted estimate

Twelve studies reported hospital mortality [19–30]. Four studies extracted data on hospital mortality from the ELSO registry [20, 21, 23, 25]. Due to overlapping study periods, we included the largest study [25] in the meta-analysis. Pooled hospital mortality of nine studies among 420 patients was 60% (95% CI 54–67%) (Fig. 3). There was low heterogeneity among the studies (*I*^2^ 6%); however, the CI was large (95% CI 0–67%). We identified two studies that were influential [25, 30] (Additional file 1: Figure S3). After excluding these two studies, the pooled hospital mortality among 66 patients was 50% (95% CI 37–63%) (Additional file 1: Figure S4 and Table S6).

### Pre-ECMO characteristics and secondary outcomes

Reporting on pre-ECMO variables and complications was inconsistent across the included studies. Therefore, data were not pooled. All patients had severe acute respiratory distress syndrome (ARDS), as reflected by PaO_2_/FiO_2_ ratio or oxygenation index (Table 2). Eight studies reported days on mechanical ventilation before ECMO was initiated, showing a range from several hours to 10 days [19–21, 23, 24, 26, 27, 29]. The total duration of ECMO varied between 1 and 72 days. Only two studies reported severity of illness scores prior to ECMO [24, 28]. However, different scores were used, making a comparison between both studies impossible. Surprisingly, scarce data on cancer type and stage, details of HSCT trajectory, and cancer treatment prior to PICU admission were provided.

**Table 2:** Pre- and on-ECMO characteristics, adverse events during the ECMO course, and outcomes

Study	Days of mechanical ventilation prior to ECMO Mean (SD) or median (IQR)	PaO_s_:FiO_2_ before start of ECMO Mean (SD) or median (IQR)	OI before start ECMO	Days on ECMO Mean (SD) or median (IQR)	Type of cannulation, *n* (%)					Mortality, *n* (%)		Adverse events, *n* (%)				
					VV	VA	VV → VA	VA → VVV	other	During ECMO run	Hospital	Major haemorrhage	Mechanical	Infections	Central nervous system	Renal
Lindén et al. 1999 [19]	5.0 (1.0–9.8)	56.5 (41.8–75.8)	NR	17.5 (9.5–36.8)	4 (100%)					1/4 (25%)	1/4 (25%)	0	1 (25%)	1 (25%)	0	0
Gupta et al. 2008 [20]	NS	NS	NR	NR	NR					NR	44/60 (73%)	NR	NR	NR	NR	NR
Gow et al. 2009 [21]	2.7 (0.9–5.3)	53.0 (42.0–62.0)	52.0 (39.3–70.0)	6.1 (2.8–12.3)	28 (26.2%)	67 (62.6%)	9 (8.4%)	1 (0.9%)	2 (1.9%)	62/107 (58%)	70/107 (65.4%)	44 (41.1%)	46 (43.0%)	40 (37.3%)	28 (26.1%)	67 (62.6%)
Meister et al. 2009 [22]	NR	NR	NR	14.0 (3.5)	3 (75%)	1 (25%)				2/4 (50%)	2/4 (50%)	1 (25%)	0	0	0	0
Di Nardo et al. 2014 [23]	2.7 (range 1.7–7.4)	NR	NR	7.7 (3.8–15.6)	8	17	3			14/15 (93%)	14/15 (93%)	NR	NR	NR	NR	NR
Smith et al. 2016 [24]	0.3 (0.1–1)	NR	19 (9.5–44.5)	5.0 (3.9–6.7))	1 (12%)	8 (88%)				5/9 (66%)	5/9 (66%)	5 (55%)		2 (22%)	1 (11%)	6 (67%)
Bailly et al. 2017 [25]	NR	NR	NR	NR	NR					NR	101/161 (62.7%)	NR	NR	NR	NR	NR
Cortina et al. 2018 [26]	3 (range 0.4–14)	47 (range 32–67)	NR	14 (range 2–24)	7 (77.8%)	2 (22.2%)				4/9 (44.4%)	5/9 (56%)	4 (44.4%)				3 (33.3%)
Maue et al. 2019 [27]	NS	NR	NS	NS	2 (40%)	3 (60%)				4/5 (80%)	5/5 (100%)	NS		0		NS
Steppan et al. 2020 [28]	NR	NR	NS	NS	NS					NS	6/16 (37.5%)				NS	NS
Ranta et al. 2020 [29]	4.5 (2.0–8.0)	NR	NR	15 (range 1–72)	4 (33.3%)	4 (33.3%)	1 (8.3%)	1 (8.3%)	2 (16.7%)	4/12 (33.3%)	6/12 (50%)	4 (33.3%)			2 (16.7%)	
Coleman et al. 2020 [30]	NR	NR	NR	5 (1–15; leukaemia); 2 (0–6; lymphoma); 2 (0–11; other)	NR					NR	126/200 (63%)	NR	NR	NR	NR	NR
Friedman et al. 2020 [31]	NR	NR	NR	NR	NR					13/23 (57%)	NR	NR	NR	NR	NR	NR

*ECMO* extracorporeal membrane oxygenation, *IQR* interquartile range, *NR* not reported, *NS* not specified for oncology patients, *PICU* pediatric intensive care unit, *SD* standard deviation, *VA* venoarterial, *VV* venovenous, *VV* → *VA* VV converted to VA, *VA* → *VV* VA converted to VV

Eight studies included data for complications [19, 21, 22, 24, 26–29] (Table 2). In view of the inconsistent reporting of these outcomes across the included studies, the adverse events were not pooled. Sixty-nine (36.5%) of 189 patients in nine studies had major hemorrhages. In 49 patients (26%), new infections during ECMO therapy were reported. One hundred and nine patients (57.7%) required renal replacement therapy on ECMO. The number of circuit-associated or cannula-associated complications was low.

### Risk of bias

Of the 13 studies, 8 had a total Newcastle–Ottawa Scale score < 7 (Additional file 1: Table S7) [19, 21–24, 26, 27, 29]. None of the studies reported comparative clinical data from unexposed groups. In 11 studies, the population was representative or somewhat representative of the average population. In all studies, the assessment of outcome was confirmed with medical records or linked through a database and the follow-up of the patients was considered adequate.

Egger’s test and funnel plots showed some, non-significant, evidence of publication bias (Additional file 1: Figures S5 and S6, Table S8). A summary of the GRADE assessment for certainty of evidence is provided in Additional file 1: Table S9.

## Discussion

To our knowledge, our systematic review is the first in which the results of ECMO in pediatric oncology patients are described. As overall pediatric cancer survival has improved and increasingly complex patients are successfully supported with ECMO, interest has grown concerning ideal use of ECMO in this population.

A recent large pediatric registry study of 9194 children between 2004 and 2013 who were supported on ECMO reported an overall final discharge mortality of 44% [30]. The pooled mortality (during ECMO 55% and in hospital 60%) in our meta-analysis is higher, but the mortality rates varied widely from 25 to 93%. In addition, of the 625 patients identified, we only included mortality data from about 50% of these patients due to overlap between the included studies. We therefore should exercise caution in interpreting the results of our study. The higher mortality found in our meta-analysis may be explained by reduced baseline cardiopulmonary reserves due to treatment toxicities, prolonged recovery and vulnerability to all ECMO complications of pediatric cancer patients. Additionally, the inclusion of HSCT patients in a part of the included studies may have resulted in high mortality rates as HSCT has been identified as an independent risk factor for mortality in previous studies [32]. Presence of sepsis, acidosis, multi-organ dysfunction and higher severity of illness scores prior to ECMO are risk factors for mortality and are more common in oncology and HSCT patients [21, 33, 34]. Only a few studies included patients with non-respiratory indications for the initiation of ECMO, including sepsis, cardiac failure, and extracorporeal cardiopulmonary resuscitation. A recent meta-analysis showed a cumulative pooled estimate survival of 55% in septic children requiring ECMO [18]. However, due to small patient numbers, we were not able to conduct subgroup analyses to determine pooled mortality rates for the patient groups included in our study. The lack of granular data on all these factors in the included studies emphasizes the need for prospective studies to enable a more detailed analysis identifying risk factors for poor outcome.

In a recent systematic review, we showed that children with an underlying malignancy who require invasive mechanical ventilation have a mortality rate of 24% [35]. All of the children receiving ECMO in our present review had severe ARDS with median PaO_2_/FiO_2_ ratios below 100 or median OIs between 19 and 52 prior to start of ECMO. There are several studies that have shown an independent association of OI > 40 with higher mortality [36, 37]. In addition, a recent review on ventilation parameters before initiation of ECMO in general pediatric patients who required ECMO for respiratory indications showed that both OI and duration of mechanical ventilation before ECMO were independently associated with in-hospital mortality [38]. The severe degree of oxygenation disturbances prior to ECMO reported in the included studies may reflect a reluctance among treating physicians to either refer for or place such children on ECMO leading to it use as a last resort. Future studies examining pre-ECMO data and mechanical ventilation parameters in a more granular manner will help in clinical decision making and counseling. Whether earlier application of ECMO may improve outcome of these patients is extremely important. A recent systematic review and meta-analysis of ECMO in adults with severe ARDS showed that compared with conventional mechanical ventilation, the use of ECMO was associated with reduced 60-day mortality [39]. However, ECMO was also associated with a moderate risk of major bleeding. There are no studies comparing ECMO with conventional ventilation in children with PARDS. We cannot reach robust conclusions yet on ECMO timing in children based on the available evidence. Thus, caution should be used when evaluating ECMO candidacy.

The use of ECMO in patients with cancer poses considerable challenges. Patients with malignancies often have abnormal myeolopoiesis either as a consequence of the underlying malignancy or its treatment that results in thrombocytopenia and leukopenia with or without neutropenia. These patients are often also coagulopathic and the need for systemic anticoagulation increases the risk of bleeding. We found an ECMO-related complication rate of 65% which is comparable to the rate of 66% reported in a mixed group of children receiving ECMO [40]. Bleeding complications were somewhat higher to that found in children without oncologic disease supported on ECMO in a recent ELSO report [41]. Importantly, despite the high prevalence of cytopenia and immunosuppressive therapy among these patients, the incidence of nosocomial infection was similar to that seen in immunocompetent children. However, data on the presence of leukopenia and lower platelet counts were lacking in the majority of the included studies. Therefore, the incidence of cytopenia and the association with complications among the included patients could not be determined. These data and the increasing reports of children with malignancies in international ECMO registries suggest an underlying malignancy should not be considered a contraindication to ECMO.

Although this is the first systematic review carried out on children with cancer on ECMO, it has some limitations. The majority of studies included used registry data from which it is impossible to capture relevant, granular data. In addition, due to inconsistent reporting, we were not able to analyze certain factors (e.g., underlying malignancy and staging, treatment response, cancer treatment prior to PICU admission and start of ECMO, role of HSCT, presence of neutropenia) which are important for clinical decision making. In addition, the indications for both the initiation and discontinuation of ECMO were often not defined, and none of the studies provided risk adjustment for severity of illness or described the outcome of matched children in whom ECMO was not used. Moreover, there is a marked heterogeneity in the oncology patient population. However, due to the lack of granularity of data on underlying malignancies and the small patient numbers, data are insufficient to conduct subgroup analysis. Therefore, the results of this study may not be generalizable to individual oncology patients. The review also included older studies that may not reflect survival in children with oncological diagnoses treated in the modern era. Lastly, another potential limitation of our study was the selection strategy used to avoid overlap between study subjects in registry studies by extraction period. By including the largest study only in case of overlapping study periods may have led to the exclusion of more than necessary non-overlapping subjects for the meta-analysis resulting in a smaller sample size. However, we found that this was a transparent and reliable approach to avoid overlap, which may have introduced even greater bias. In addition, an overlap between centers from the USA participating in more than one registry (ELSO, PEDECOR, and PHIS database) could not be excluded. However, there were only slightly overlapping time periods [28] and different inclusion criteria [30].

Characterization of illness severity, primary malignancy and malignancy status, therapies that were administered (chemotherapy, radiotherapy, surgery, immunotherapy), presence of leukopenia before ECMO, and the ultimate outcome from the malignancy were not reported in the majority of the included studies. It is likely that a large part of the observed range in heterogeneity can be attributed to differences in these variables that are difficult to control for without access to individual patient data.

The risk of bias is difficult to accurately define in meta-analyses of observational studies [42] and is made more challenging because pre-registration and protocol preparation are not mandatory. As a result, data from unpublished studies or partly unpublished results cannot be identified. This may lead to an increased risk of publication bias and other reporting biases such as selective outcome reporting. In addition, only retrospective studies were identified. These have inherent limitations such selection bias and missing data. However, observational studies provide valuable supplementary information regarding safety and long-term outcomes of interventions. Their results might be more directly applicable to a general population as they are conducted under a more real-life setting than RCTs, which usually involve very restricted populations treated with highly standardized care.

Despite the limitations, this review provides an important summary of the published outcomes and complications of ECMO used to support children with oncological diagnoses. This could be used to enlighten discussions between critical care providers and oncologists when faced with a child with cancer who has pulmonary or cardiac dysfunction refractory to conventional care.

## Conclusion

Overall, aligned with the trend towards improving oncological outcomes, ECMO could represent a viable and ethically justifiable rescue therapy for some of these patients despite the higher mortality compared to the general PICU population. Future studies are needed to refine patient selection and optimize the timing of intervention, and to define patients for whom ECMO may provide benefit or harm [43]. Until these new results become available, data on use of ECMO in this vulnerable patient population remain inconclusive.

## Supplementary Information

**Additional file 1: Table S1.** PRISMA-P checklist. **Table S2.** Search strategy. **Table S3.** Patient characteristics of included studies. **Table S4.** Indications for ECMO and related hospital mortality. **Figure S1.** Influence analyses of studies reporting on ECMO mortality. **Figure S2.** Estimated pooled ECMO mortality when influential studies are excluded. **Table S5.** Summary result meta-analysis ECMO mortality. **Figure S3.** Influence analyses studies reporting hospital mortality. **Figure S4.** Estimated pooled hospital mortality when influential studies are excluded. **Table S6.** Summary result meta-analysis hospital mortality. **Table S7.** Quality Assessment Studies. **Figure S5.** Funnel plot of studies reporting on ECMO mortality. **Figure S6.** Funnel plot of studies reporting on hospital mortality. **Table S8.** Egger’s test result for publication bias. **Table S9.** GRADE assessment.

## Data Availability

All data generated or analyzed during this study are included in the published studies and their additional information files.

## Abbreviations

ARDS: Acute respiratory distress syndrome
CI: Confidence interval
ECMO: Extracorporeal membrane oxygenation
ELSO: Extracorporeal Life Support Organization
GRADE: Grading of Recommendations, Assessments, Developments and Evaluations
HSCT: Hematopoietic stem cell transplantation
PICU: Pediatric intensive care unit
PRISMA: Preferred Reporting Items for Systematic Reviews and Meta-Analyses
