# Supplementary material for: Role of extracorporeal membrane oxygenation in pediatric cancer patients: a systematic review and meta-analysis of observational studies
Source: Ann Intensive Care. 2022 Jan 29;12:8. doi: 10.1186/s13613-022-00983-0 (PMC8800958; doi:10.1186/s13613-022-00983-0)
Supplement: Supplementary file 1 — Additional file 1: Table S1. PRISMA-P checklist. Table S2. Search strategy. Table S3. Patient characteristics of included studies. Table S4. Indications for ECMO and related hospital mortality. Figure S1. Influence analyses of studies reporting on ECMO mortality. Figure S2. Estimated pooled ECMO mortality when influential studies are excluded. Table S5. Summary result meta-analysis ECMO mortality. Figure S3. Influence analyses studies reporting hospital mortality. Figure S4. Estimated pooled hospital mortality when influential studies are excluded. Table S6. Summary result meta-analysis hospital mortality. Table S7. Quality Assessment Studies. Figure S5. Funnel plot of studies reporting on ECMO mortality. Figure S6. Funnel plot of studies reporting on hospital mortality. Table S8. Egger’s test result for publication bias. Table S9. GRADE assessment. [file 13613_2022_983_MOESM1_ESM.docx]

**Supplementary material**

**Role of extracorporeal membrane oxygenation in pediatric cancer patients:**

**a systematic review and meta-analysis of observational studies.**

The POKER (PICU Oncology Kids in Europe Research group) research consortium of ESPNIC (European Society of Paediatric Neonatal Intensive Care)

**Contents**

| Table S1. PRISMA-P checklist | Page 2-3 |
| --- | --- |
| Table S2. Search strategy | Page 4-5 |
| Table S3. Patient characteristics of included studies  Table S4. Indications for ECMO and related hospital mortality  Figure S1 Influence analyses of studies reporting on ECMO mortality  Figure S2. Estimated pooled ECMO mortality when influential studies are excluded  Table S5. Summary result meta-analysis ECMO mortality  Figure S3 Influence analyses studies reporting hospital mortality  Figure S4. Estimated pooled hospital mortality when influential studies are excluded  Table S6. Summary result meta-analysis hospital mortality  Table S7. Quality Assessment Studies  Figure S5. Funnel plot of studies reporting on ECMO mortality  Figure S6. Funnel plot of studies reporting on hospital mortality  Table S8. Egger’s test result for publication bias  Table S9. GRADE assessment | Page 6  Page 7  Page 8  Page 9  Page 9  Page 10  Page 11  Page 11  Page 12-13  Page 14  Page 14  Page 15  Page 16 |
|  |  |

**Table S1. PRISMA-P (Preferred Reporting Items for Systematic review and Meta-Analysis Protocols) 2015 checklist: recommended items to address in a systematic review protocol.**

| **Section and topic** | **Item No** | **Checklist item** | **Inclusion**  **(Section or page number)** |
| --- | --- | --- | --- |
| **ADMINISTRATIVE INFORMATION** | | | |
| Title:   - Identification - Update | 1a  1b | Identify the report as a protocol of a systematic review  If the protocol is for an update of a previous systematic review, identify as such | Title  n/a |
| Registration | 2 | If registered, provide the name of the registry (such as PROSPERO) and registration number | n/a |
| Authors:   - Contact - Contributions | 3a  3b | Provide name, institutional affiliation, e-mail address of all protocol authors; provide physical mailing address of corresponding author  Describe contributions of protocol authors and identify the guarantor of the review | Title page  Page 14 |
| Amendments | 4 | If the protocol represents an amendment of a previously completed or published protocol, identify as such and list changes; otherwise, state plan for documenting important protocol amendments | n/a |
| Support:   - Sources - Sponsor - Role of sponsor or funder | 5a  5b  5c | Indicate sources of financial or other support for the review  Provide name for the review funder and/or sponsor  Describe roles of funder(s), sponsor(s), and/or institution(s), if any, in developing the protocol | No funding  n/a  n/a |
| **INTRODUCTION** | | | |
| Rationale | 6 | Describe the rationale for the review in the context of what is already known | Page 5 |
| Objectives | 7 | Provide an explicit statement of the question(s) the review will address with reference to participants, interventions, comparators, and outcomes (PICO) | Page 5 |
| **METHODS** | | | |
| Eligibility criteria | 8 | Specify the study characteristics (such as PICO, study design, setting, time frame) and report characteristics (such as years considered, language, publication status) to be used as criteria for eligibility for the review | Page 6 |
| Information sources | 9 | Describe all intended information sources (such as electronic databases, contact with study authors, trial registers or other grey literature sources) with planned dates of coverage | Page 6 |
| Search strategy | 10 | Present draft of search strategy to be used for at least one electronic database, including planned limits, such that it could be repeated | Supplemental Table 2 |
| Study records:   - Data management - Selection process      - Data collection process   Data items | 11a  11b  11c  12 | Describe the mechanism(s) that will be used to manage records and data throughout the review  State the process that will be used for selecting studies (such as two independent reviewers) through each phase of the review (that is, screening, eligibility and inclusion in meta-analysis)  Describe planned method of extracting data from reports (such as piloting forms, done independently, in duplicate), any processes for obtaining and confirming data from investigators  List and define all variables for which data will be sought (such as PICO items, funding sources), any pre-planned data assumptions and simplifications | Page 6-7  Page 6-7  Page 7  Page 7 |
| Outcomes and prioritization | 13 | List and define all outcomes for which data will be sought, including prioritization of main and additional outcomes, with rationale | Page 7 |
| Risk of bias in individual studies | 14 | Describe anticipated methods for assessing risk of bias of individual studies, including whether this will be done at the outcome or study level, or both; state how this information will be used in data synthesis | Page 7-8 |
| Data synthesis | 15a  15b  15c  15d | Describe criteria under which study data will be quantitatively synthesized  If data are appropriate for quantitative synthesis, describe planned summary measures, methods of handling data and methods of combining data from studies, including any planned exploration of consistency (such as I2, Kendall’s τ)  Describe any proposed additional analyses (such as sensitivity or subgroup analyses, meta-regression)  If quantitative synthesis is not appropriate, describe the type of summary planned | Page 7-8  Page 7-8  n/a |
| Meta-bias(es) | 16 | Specify any planned assessment of meta-bias(es) (such as publication bias across studies, selective reporting within studies) | Page 7-8 |
| Confidence in cumulative evidence | 17 | Describe how the strength of the body of evidence will be assessed (such as GRADE) | GRADE |

*From: Shamseer L, Moher D, Clarke M, Ghersi D, Liberati A, Petticrew M, Shekelle P, Stewart L, PRISMA-P Group. Preferred reporting items for systematic review and meta-analysis protocols (PRISMA-P) 2015: elaboration and explanation. BMJ. 2015 Jan 2;349(jan02 1):g7647.*

**Table S2. Search strategy**

| **Database: PubMed**  Data searched: till 30 September 2020  Records retrieved: 704  **Child**  (adolescent[MeSH] OR child[MeSH] OR "Infant"[Mesh] OR infan*[Title/Abstract] OR newborn*[Title/Abstract] OR new-born*[Title/Abstract] OR perinat*[Title/Abstract] OR neonat*[Title/Abstract] OR baby[Title/Abstract] OR babies[Title/Abstract] OR toddler*[Title/Abstract] OR minor[Title/Abstract] OR minors*[Title/Abstract] OR boy[Title/Abstract] OR boys[Title/Abstract] OR boyfriend[Title/Abstract] OR boyhood[Title/Abstract] OR girl*[Title/Abstract] OR kid[Title/Abstract] OR kids[Title/Abstract] OR child[Title/Abstract] OR children[Title/Abstract] OR childhood[Title/Abstract] OR schoolchild*[Title/Abstract] OR school child*[Title/Abstract] OR adolescen*[Title/Abstract] OR juvenil*[Title/Abstract] OR youth*[Title/Abstract] OR teen*[Title/Abstract] OR underage*[Title/Abstract] OR pubescen*[Title/Abstract] OR pediatric*[Title/Abstract] OR paediatric*[Title/Abstract] OR peadiatric*[Title/Abstract] OR prematur*[Title/Abstract] OR preterm*[Title/Abstract])  **Cancer**  (leukemi*[Title/Abstract] OR leukaemi*[Title/Abstract] OR ALL[Title/Abstract] OR AML[Title/Abstract] OR lymphom*[Title/Abstract] OR hodgkin*[Title/Abstract] OR non-hodgkin*[Title/Abstract] OR T-cell[Title/Abstract] OR B-cell[Title/Abstract] OR sarcom*[Title/Abstract] OR sarcoma ewings[Title/Abstract] OR ewing*[Title/Abstract] OR osteosarcom*[Title/Abstract] OR wilms[Title/Abstract] OR nephroblastom*[Title/Abstract] OR neuroblastom*[Title/Abstract] OR rhabdomyosarcom*[Title/Abstract] OR teratom*[Title/Abstract] OR hepatom*[Title/Abstract] OR hepatoblastom*[Title/Abstract] OR PNET[Title/Abstract] OR medulloblastom*[Title/Abstract] OR metasta*[Title/Abstract] OR neuroectodermal tumor*[Title/Abstract] OR retinoblastom*[Title/Abstract] OR meningiom*[Title/Abstract] OR gliom*[Title/Abstract] OR paraneoplastic[Title/Abstract] OR cancer*[Title/Abstract] OR oncolog*[Title/Abstract] OR oncogen* [Title/Abstract] OR neoplasm*[Title/Abstract] OR carcinom*[Title/Abstract] OR tumor[Title/Abstract] OR tumors[Title/Abstract] OR tumour*[Title/Abstract] OR malignan*[Title/Abstract] OR hematooncologic*[Title/Abstract] OR hemato oncologic*[Title/Abstract] OR neoplasm*[Title/Abstract] OR hematolo*[Title/Abstract] OR hematopoietic*[Title/Abstract] OR stem cell[Title/Abstract] OR transplant[Title/Abstract] OR neoplasms[Mesh] OR immunocompromised*[Title/Abstract])  **ECMO**  (ECMO*[Title/Abstract]) OR "extracorporeal membrane oxygenation"[Title/Abstract] OR "extra corporeal membrane oxygenation"[Title/Abstract] OR "extra-corporeal membrane oxygenation"[Title/Abstract] OR ECLS*[Title/Abstract] OR “extracorporeal oxygenation”[Title/Abstract] OR “extra-corporeal oxygenation”[Title/Abstract] OR “extra corporeal oxygenation”[Title/Abstract] OR "extracorporeal life support"[Title/Abstract] OR "extra corporeal life support"[Title/Abstract] OR "extra-corporeal life support"[Title/Abstract] OR extracorporeal membrane oxygenation[MeSH]) |
| --- |
| **Database: Embase**  Data searched: 30 September 2020  Records retrieved: 634   \| No. \| Query \| \| --- \| --- \| \| #1 \| 'extracorporeal oxygenation'/exp \| \| #2 \| 'extracorporeal oxygenation' \| \| #3 \| 'extracorporeal membrane oxygenation' \| \| #4 \| 'extracorporeal' AND 'membrane' AND 'oxygenation' \| \| #5 \| ecmo \| \| #6 \| 'extracorporeal oxygenation'/exp OR 'extracorporeal oxygenation' OR 'extracorporeal membrane oxygenation' OR ('extracorporeal' AND 'membrane' AND 'oxygenation') OR ecmo \| \| #7 \| 'neoplasm'/exp \| \| #8 \| 'neoplasms' \| \| #9 \| 'cancer' \| \| #10 \| 'oncology' \| \| #11 \| 'neoplasm'/exp OR 'neoplasms' OR 'cancer' OR 'oncology' \| \| #12 \| 'child'/exp \| \| #13 \| 'children' \| \| #14 \| 'pediatric' \| \| #15 \| 'infant'/exp \| \| #16 \| 'infant' \| \| #17 \| 'child'/exp OR 'children' OR 'pediatric' OR 'infant'/exp OR 'infant' \| \| #18 \| ('extracorporeal oxygenation'/exp OR 'extracorporeal oxygenation' OR 'extracorporeal membrane oxygenation' OR ('extracorporeal' AND 'membrane' AND 'oxygenation') OR ecmo) AND ('neoplasm'/exp OR 'neoplasms' OR 'cancer' OR 'oncology') AND ('child'/exp OR 'children' OR 'pediatric' OR 'infant'/exp OR 'infant') \| |
| **Database: CINAHL**  Data searched: 30 September 2020  Records retrieved: 19  S1 MH neoplasms OR TI cancer OR AB cancer OR TI oncology OR AB oncology OR TI oncological OR AB oncological  S2 MH extracorporeal membrane oxygenation OR TI ecmo OR AB ecmo OR TI "extracorporeal membrane oxygenation" OR AB "extracorporeal membrane oxygenation"  S3 MH child OR TI child OR AB child OR TI pediatric OR AB pediatric  S4 (MH child OR TI child OR AB child OR TI pediatric OR AB pediatric) AND (S1 AND S2 AND S3) |

**Table S3. Patient characteristics of included studies**

| **Study** | **Underlying malignancy, n (%)** | | | | | **HSCT, n (%)** | **Age, median (IQR), yrs** | **Indication for ECMO** |
| --- | --- | --- | --- | --- | --- | --- | --- | --- |
|  | **Leukemia**  **&**  **Lymphoma** | **Solid**  **tumors** | **Brain &**  **Spinal Cord**  **tumors** | **Others** | **Non-malignant or not reported** |  |  |  |
| Lindén et al., 1999 [19] | 3 (75%) |  | 1 (25%) |  |  | NR | 6 (5.3-11.3) | PCP pneumonia with PaO_2_:FiO_2_ <150 and intrapulmonary venous admixture (shunt fraction) > 30% |
| Gupta et al., 2008 [20] | 49 (27%) |  |  | 11 ns (6%) | 123 (67%) | 17 (9%) | 3.1 (0.9-10.2) | Respiratory failure |
| Gow et al., 2009 [21] | 73 (68%) | 34 (32%) |  |  |  | 0 | 3.71 (1.7-11.6) | Respiratory or cardiac failure |
| Meister et al., 2009 [22] | 4 (100%) |  |  |  |  | NR | 14 (3.6-15.0) | Respiratory failure (ARDS) |
| Di Nardo et al., 2014 [23] | 13 (45%) |  |  | 2 (7%) | 14 (48%) | 29 (100%) | 5 (range 1.2-11.9) | Pulmonary support (80%), cardiac support (13%) and during CPR (6%) |
| Smith et al., 2016 [24] | 7 (78%) | 2 (22%) |  |  |  | 1 (11.1%) | 9 (5-11) | Neutropenic sepsis patients with both potentially curable malignancy and reversible shock deemed refractory to all other forms of pharmacological and ventilator management |
| Bailly et al., 2017 [25] | NR | NR | NR | NR | NR | NR | NR | Primary pulmonary diagnosis |
| Cortina et al, 2018 [26] | 9 (100%) |  |  |  |  | NR | 14 (range 1-18) | Acute respiratory failure: Pulmonary infections (78%), TRALI (11%), leukemic infiltration (11%) |
| Maue et al., 2019 [27] | 5 (71%) |  |  |  | 2 (29%) | 2 (29%) | NR | Acute respiratory failure (72%), septic shock (14%), cardiac failure (14%) |
| Steppan et al., 2020 [28] | 10 (56%) | 6 (33%) |  |  | 2 (11%) | 8 (44%) | 9.44 (5.99) | Respiratory failure (66.7%), sepsis (16.7%), cardiac failure (11.1%), combined respiratory and cardiac failure (5.6%) |
| Ranta et al, 2020 [29] | 12 (100%) |  |  |  |  | NR | 2.9 (1.5-7.1) | Respiratory failure (83%), (E)CPR (17%) |
| Coleman et al, 2020 [30] | 117 (59%) | 28 (14%) | 12 (6%) | 43 (22%) |  |  | NR | NR |
| Friedman et al, 2020 [31] | NR | NR | NR | NR | NR | NR | NR | Respiratory failure |

(E)CPR = (extracorporeal) cardiopulmonary resuscitation; ECMO = Extracorporeal membrane oxygenation; ELSO registry = Extracorporeal Life Support Organization registry including >145 centers worldwide; H(S)CT = hematopoietic (stem) cell transplantation; PCP = *Pneumocystis carinii* pneumonia; NR = not reported; ns = not specified; Retro = retrospective; TRALI = transfusion-related acute lung injury; SD = standard deviation; IQR = interquartile range; yrs = years

**Table S4. Indications for ECMO and related hospital mortality**

| **Study** | **Indication for ECMO** | | | |  | **Hospital mortality** |  | |  | |  | |
| --- | --- | --- | --- | --- | --- | --- | --- | --- | --- | --- | --- | --- |
|  | **Respiratory failure** | **Sepsis** | **Cardiac support** | **ECPR** |  | **Respiratory failure** | **Sepsis** | **Cardiac support** | | **ECPR** | |  |
| Lindén et al., 1999 [19] | 4/4 (100%) |  |  |  |  | 1/4 (25%) |  |  | |  | |  |
| Gupta et al., 2008 [20] | 60/60 (100%) |  |  |  |  | 44/60 (73%) |  |  | |  | |  |
| Gow et al., 2009 [21] | 86/107 (80%) |  | 14/107 (13%) | 7/107 (7%) |  | 55/86 (64%) |  | 10/14 (71%) | | 5/7 (71%) | |  |
| Meister et al., 2009 [22] | 4/4 (100%) |  |  |  |  | 2/4 (50%) |  |  | |  | |  |
| Di Nardo et al., 2014 [23] | 12/15 (80%) |  | 2 (13%) | 1 (7%) |  | 11/12 92%) |  | 2/2 (100%) | | 1/1 (100%) | |  |
| Smith et al., 2016 [24] |  | 9/9 (100%) |  |  |  |  | 5/9 (56%) |  | |  | |  |
| Bailly et al., 2017 [25] | 161/161 (100%) |  |  |  |  | 101/1616 (63%) |  |  | |  | |  |
| Cortina et al, 2018 [26] | 9/9 (100%) |  |  |  |  | 6/9 (67%) |  |  | |  | |  |
| Maue et al., 2019 [27] | 1/5 (20%) | 3/5 (60%) | 1/5 (20%) |  |  | 1/1 (100%) | 3/3 (100%) | 1/1 (100%) | |  | |  |
| Steppan et al., 2020 [28] | NS | NS | NS | NS |  | NS | NS | NS | | NS | |  |
| Ranta et al, 2020 [29] | 10/12 (83%) |  |  | 2/12 (17%) |  |  | 4/10 (40%) |  | | 2/2 (100%) | |  |
| Coleman et al, 2020 [30] | NR | NR | NR | NR |  | NR | NR | NR | | NR | |  |
| Friedman et al, 2020 [31] | 23/23 (100%) |  |  |  |  | 13/23 (56%)^*^ |  |  | |  | |  |
| ^*^Only PICU mortality reported  ECPR = extracorporeal cardiopulmonary resuscitation; NR = not reported; NS = numbers not specified for oncology patients | | | | | | | | | | | |  |


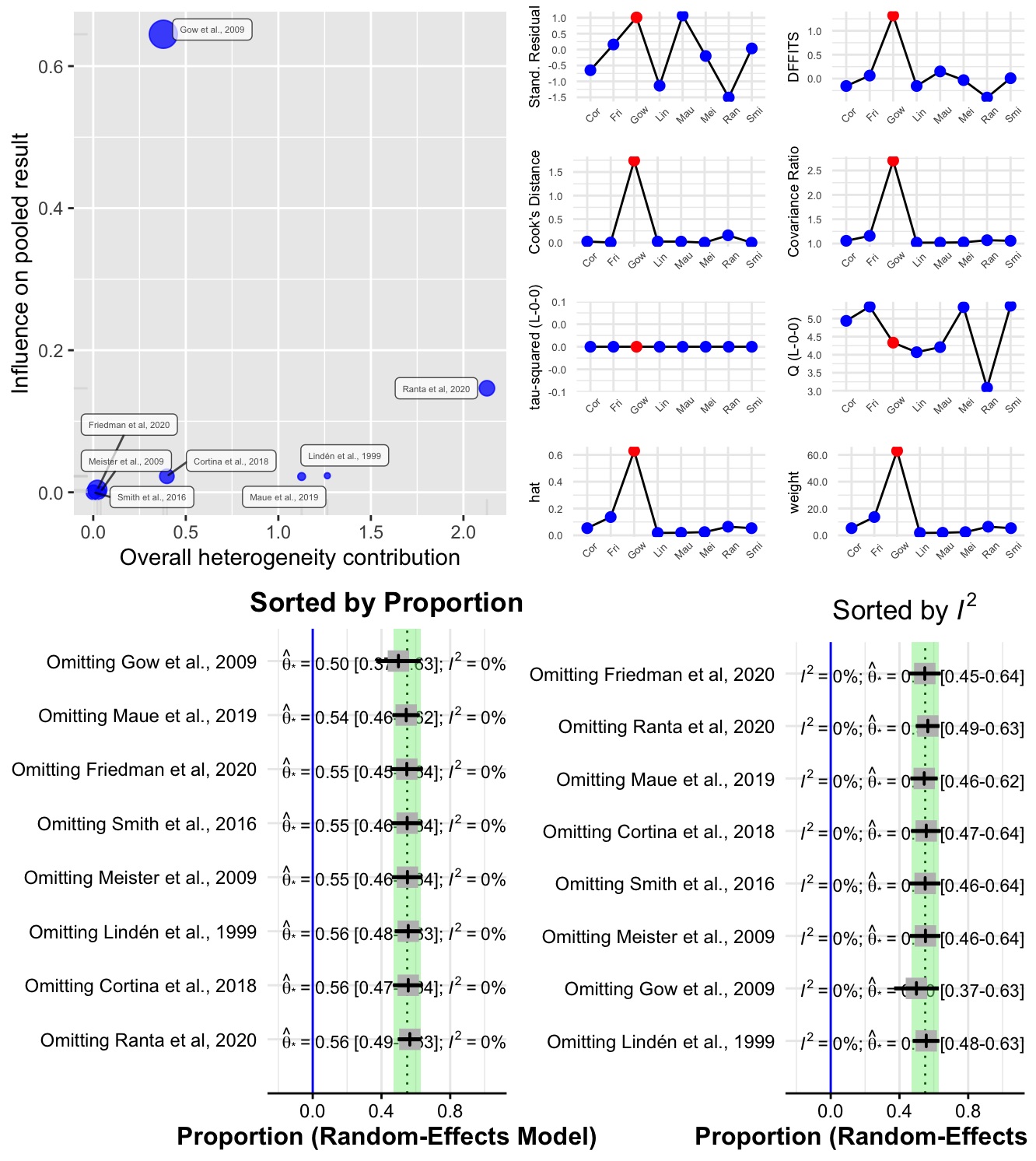


Figure S1. Influence analyses of studies reporting on ECMO mortality


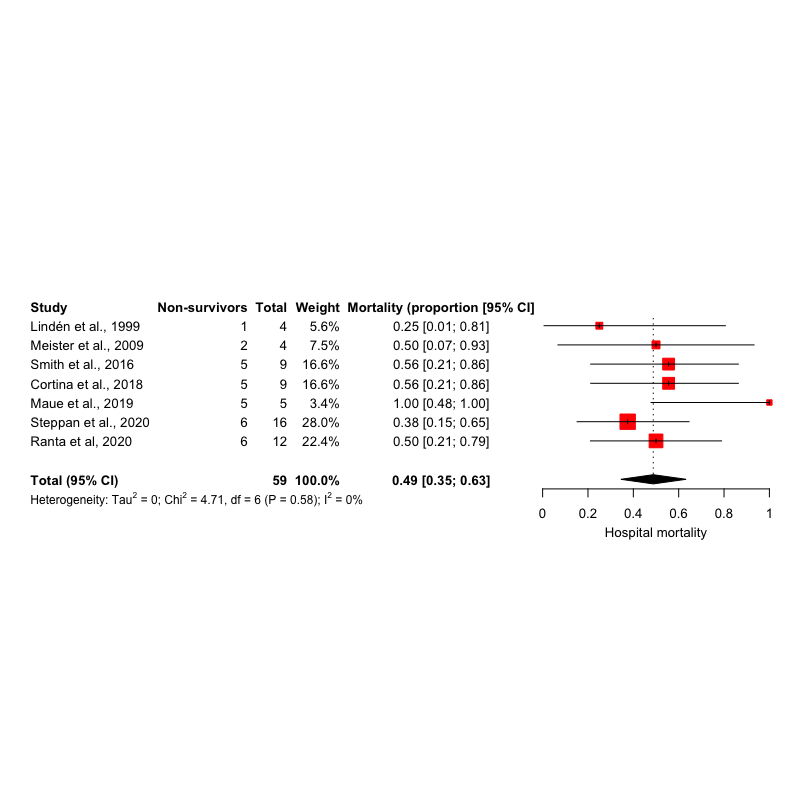


Figure S2. Estimated pooled ECMO mortality when influential studies are excluded

**Table S5. Summary result meta-analysis ECMO mortality**

| **Analysis** | **Pooled ECMO mortality** | **95% CI** | **I^2^** | **95% CI** |
| --- | --- | --- | --- | --- |
| Main analysis | 55% | 47-63% | 0 | 0-64.8% |
| Infl cases removed^1^ | 49% | 35-63% | 0 | 0-67.6% |

^1^Removed as influential: Gow et al., 2009


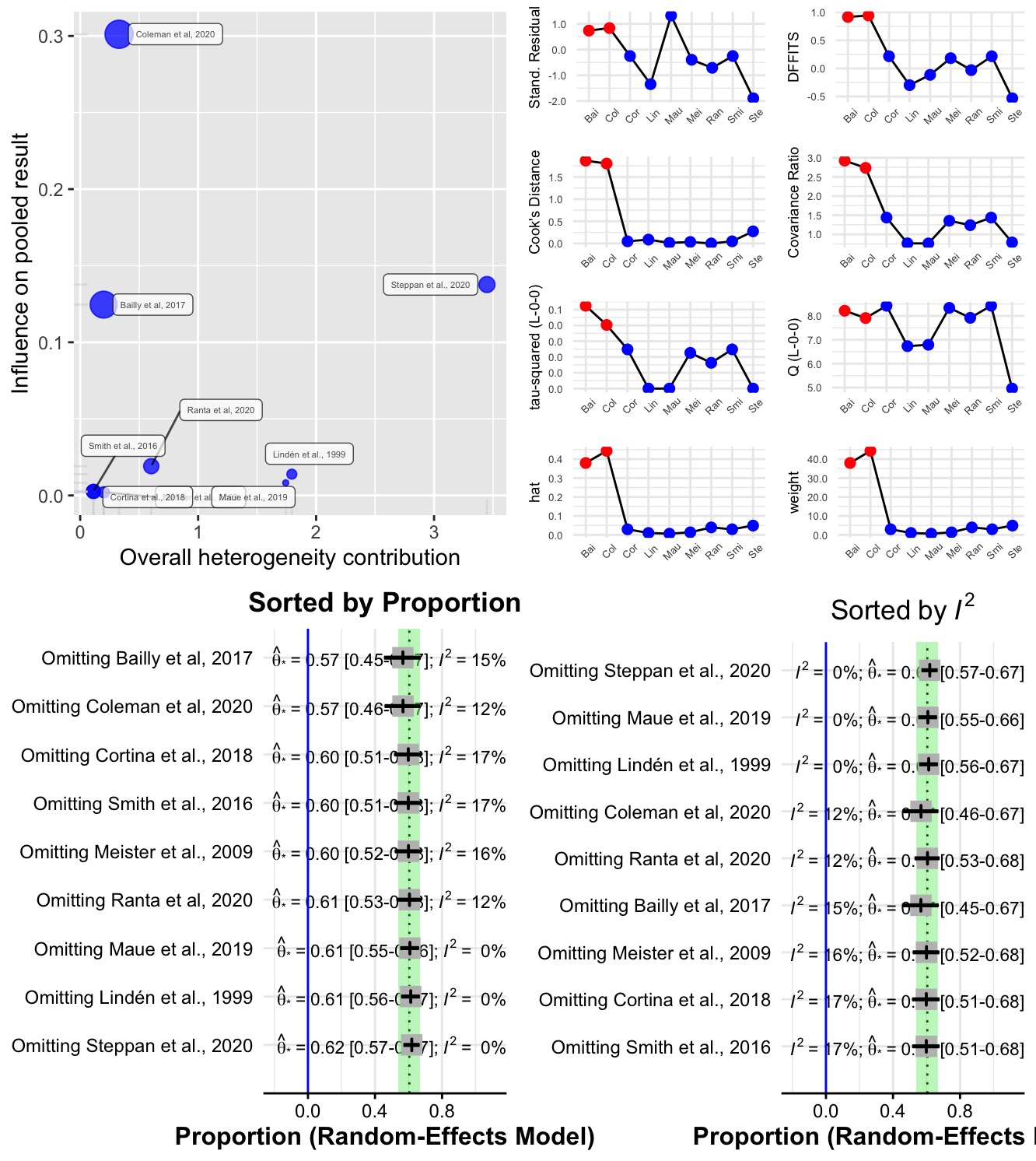


Figure S3 Influence analyses studies reporting hospital mortality


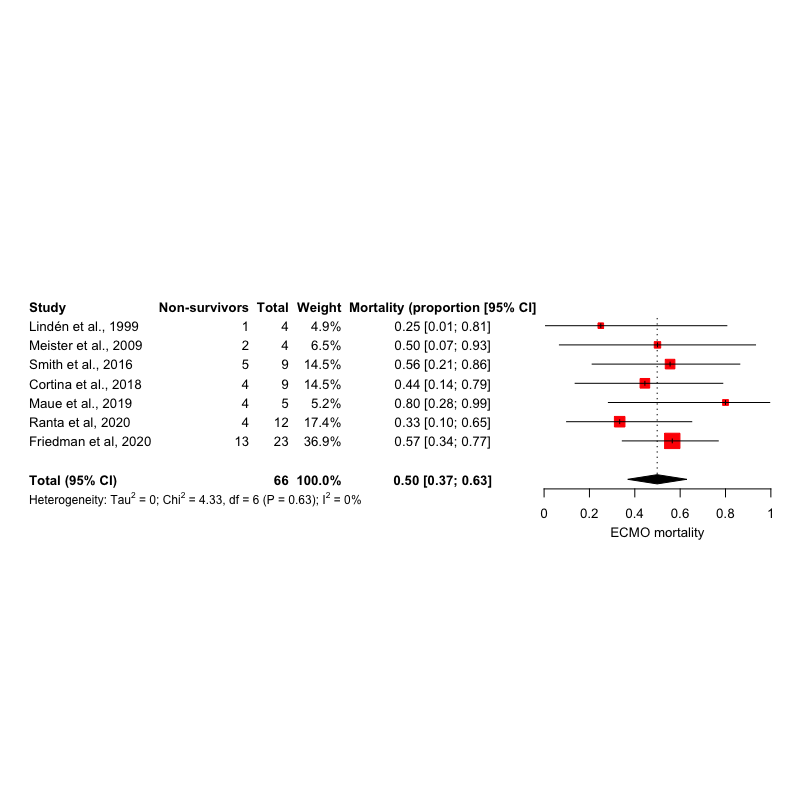


Figure S4. Estimated pooled hospital mortality when influential studies are excluded

**Table S6. Summary result meta-analysis hospital mortality**

| **Analysis** | **Pooled hospital mortality** | **95% CI** | **I^2^** | **95% CI** |
| --- | --- | --- | --- | --- |
| Main analysis | 60% | 54-67% | 0 | 0-66% |
| Infl cases removed^1^ | 50% | 37-63% | 0 | 0-71% |

^1^Removed as influential: Bailly et al., 2017; Coleman et al., 2020

**Table S7. Quality Assessment Studies**

|  | Selection | | | | Comparability | Outcome | | |  |
| --- | --- | --- | --- | --- | --- | --- | --- | --- | --- |
| Study | Representativeness of exposure | Selection of the non-exposed | Ascertainment of exposure | Outcome not present at start |  | Assessment | Follow-up length | Adequacy of follow-up | Overall score |
| Lindén et al., 1999 [19] | - | - | ★ | ★ | - | ★ | ★ | ★ | 5 |
| Gupta et al., 2008 [20] | ★ | - | ★ | ★ | ★★ | ★ | ★ | ★ | 8 |
| Gow et al., 2009 [21] | ★ | - | ★ | ★ | - | ★ | ★ | ★ | 6 |
| Meister et al., 2009 [22] | - | - | ★ | ★ | - | ★ | ★ | ★ | 5 |
| Di Nardo et al., 2014 [23] | ★ | - | ★ | ★ | - | ★ | ★ | ★ | 6 |
| Smith et al., 2016 [24] | ★ | - | ★ | ★ | - | ★ | ★ | ★ | 6 |
| Bailly et al., 2017 [25] | ★ | - | ★ | ★ | ★ | ★ | ★ | ★ | 7 |
| Cortina et al., 2018 [26] | ★ | - | ★ | ★ | - | ★ | ★ | ★ | 6 |
| Maue et al., 2019 [27] | ★ | - | ★ | ★ | - | ★ | ★ | ★ | 6 |
| Steppan et al., 2020 [28] | ★ | - | ★ | ★ | ★★ | ★ | ★ | ★ | 8 |
| Ranta et al., 2020 [29] | ★ | - | ★ | ★ | - | ★ | ★ | ★ | 6 |
| Coleman et al., 2020 [30] | ★ | - | ★ | ★ | ★ | ★ | ★ | ★ | 7 |
| Friedman et al., 2020 [31] | ★ | - | ★ | ★ | ★★ | ★ | ★ | ★ | 8 |

**Newcastle-Ottawa Quality Assessment Form for Cohort Studies**

Note: A study can be given a maximum of one star for each numbered item within the Selection and Outcome categories. A maximum of two stars can be given for Comparability.

**Selection**

1) Representativeness of the exposed cohort

a) Truly representative ***(one star)***

b) Somewhat representative ***(one star)***

c) Selected group

d) No description of the derivation of the cohort

2) Selection of the non-exposed cohort

a) Drawn from the same community as the exposed cohort ***(one star)***

b) Drawn from a different source

c) No description of the derivation of the non-exposed cohort

3) Ascertainment of exposure

a) Secure record (e.g., surgical record) ***(one star)***

b) Structured interview ***(one star)***

c) Written self-report

d) No description

e) Other

4) Demonstration that outcome of interest was not present at start of study

a) Yes ***(one star)***

b) No

**Comparability**

1) Comparability of cohorts on the basis of the design or analysis controlled for confounders

a) The study controls for age, sex and marital status ***(one star)***

b) Study controls for other factors (list) _________________________________ ***(one star)***

c) Cohorts are not comparable on the basis of the design or analysis controlled for confounders

**Outcome**

1) Assessment of outcome

a) Independent blind assessment ***(one star)***

b) Record linkage ***(one star)***

c) Self report

d) No description

e) Other

2) Was follow-up long enough for outcomes to occur

a) Yes ***(one star)***

b) No

Indicate the median duration of follow-up and a brief rationale for the assessment above:____________________

3) Adequacy of follow-up of cohorts

a) Complete follow up- all subject accounted for ***(one star)***

b) Subjects lost to follow up unlikely to introduce bias- number lost less than or equal to 20% or description of those lost suggested no different from those followed. ***(one star)***

c) Follow up rate less than 80% and no description of those lost

d) No statement


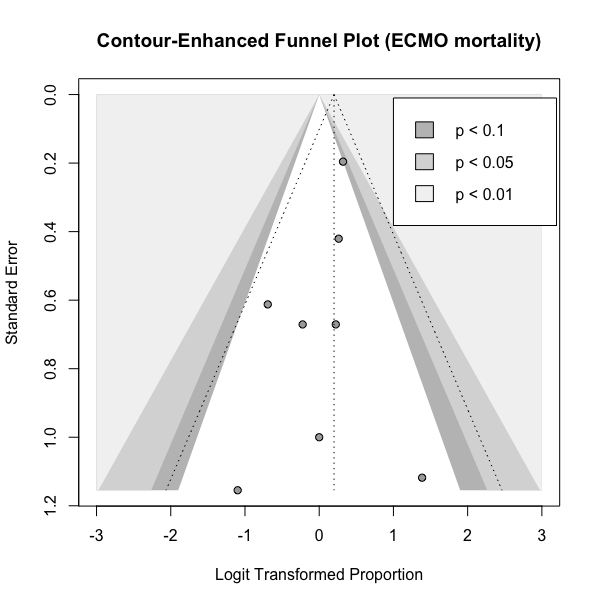


Figure S5. Funnel plot of studies reporting on ECMO mortality


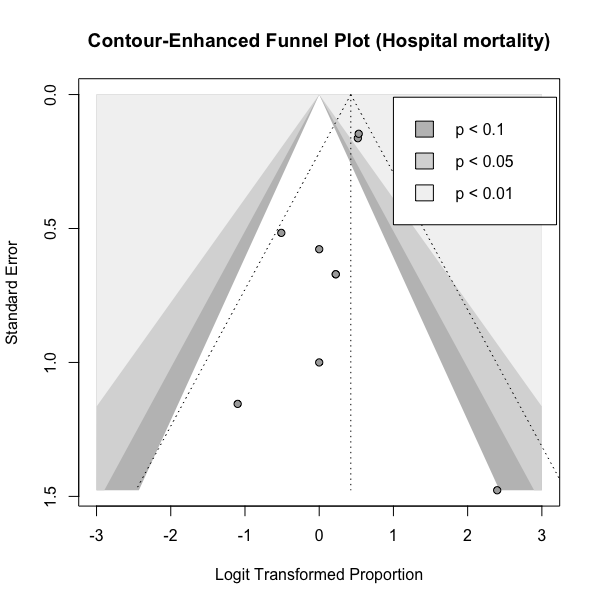


Figure S6. Funnel plot of studies reporting on hospital mortality

**Table S8. Egger’s test result for publication bias**

| **Groups** | **p-value^a^** | **p-value^b^** |
| --- | --- | --- |
| ECMO mortality | 0.31 | 0.85 |
| Hospital mortality | 0.20 | 0.33 |

^a^Before removal of influential studies; ^b^After removal of influential studies.

**Table S9. GRADE assessment of results of meta-analysis for use of ECMO in pediatric cancer patients.**

| **N^o^ of studies** | **Certainty assessment** | | | | | | |  | **Effect** | | | **Certainty** | **Importance** |
| --- | --- | --- | --- | --- | --- | --- | --- | --- | --- | --- | --- | --- | --- |
|  | **Study design** | **Risk of bias** | **Inconsistency** | **Indirectness** | **Imprecision** | **Publication bias** | **Other considerations** |  | **N^o^ of events** | **N^o^ of individuals** | **Rate (95% CI)** |  |  |
| *ECMO mortality*  9 | Observational studies | Serious^a^ | Not serious | Not serious | Not serious | Undetected | None |  | 103 | 193 | 0.54 (0.46-0.61) | ⊕⊕⊕◯  MODERATE | CRITICAL |
| *Hospital mortality*  9 | Observational studies | Serious^a^ | Not serious | Not serious | Not serious | Undetected | None |  | 259 | 424 | 0.61 (0.54-0.67) | ⊕⊕⊕◯  MODERATE | CRITICAL |
| ^a^ There was a lack of a control (non-exposed) group in all studies and a low level of comparability in the majority of the included studies | | | | | | | | | | | | | |
